# Cocreation of a Digital Psychological Intervention to Improve Mental Health Among Survivors of Breast Cancer (SerenApp): Protocol for a Usability and Pilot Randomized Controlled Trial

**DOI:** 10.2196/87151

**Published:** 2026-09-30

**Authors:** Irene Zarcos-Pedrinaci, Vanesa Ramos-García, María Padilla-Ruíz, Francisco Rivas-Ruíz, Antoni Baena, Santiago Galán, Andrea Duarte-Díaz, Lilisbeth Perestelo-Pérez, Yolanda Álvarez Pérez, Cristina Valcárcel-Nazco, Maximino Redondo Bautista, Esperanza Varela-Moreno

**Affiliations:** 1 Oncology Service, Costa del Sol University Hospital Marbella, Málaga Spain; 2 Network for Research on Chronicity, Primary Care, and Health Promotion (RICAPPS), Carlos III Health Institute Madrid Spain; 3 Instituto de Investigación Biomédica de Málaga y Plataforma en Nanomedicina – IBIMA Plataforma BIONAND Málaga Spain; 4 Instituto de Investigación Sanitaria de Canarias (IISC) Santa Cruz de Tenerife Spain; 5 Canary Islands Health Research Institute Foundation (FIISC) Santa Cruz de Tenerife Spain; 6 Research and Innovation Unit Costa del Sol University Hospital Marbella, Málaga Spain; 7 Faculty of Psychology, Education and Sport Sciences Ramon Llull University (URL) Barcelona Spain; 8 Universitat Oberta de Catalunya Barcelona Spain; 9 Department of Personality, Evaluation, and Psychological Treatment, Faculty of Psychology University of Málaga Málaga Spain

**Keywords:** breast cancer survivors, cocreation, cost-effectiveness, digital psychological intervention, effectiveness, eHealth, mental health, mobile app, protocol, user-centered design

## Abstract

**Background:**

Survivors of breast cancer frequently experience anxiety, stress, and depression during the follow-up and survivorship phases, when access to psychosocial support is often limited. Although psychological interventions have demonstrated effectiveness, their implementation in routine clinical practice remains challenging. Digital mental health interventions offer a promising alternative. However, many existing solutions show limited personalization, engagement, and long-term adherence, partly due to insufficient involvement of end users in their design.

**Objective:**

This study aims to develop and pilot-test a cocreated digital psychological intervention (SerenApp) aimed at improving psychological well-being among women during breast cancer follow-up and to evaluate its feasibility, usability, and preliminary effectiveness.

**Methods:**

This mixed methods study adopts a participatory cocreation approach structured into 3 phases. Phase 1 focuses on identifying unmet psychosocial needs and co-designing the app’s content and functionalities through workshops involving patients and health care professionals, applying design thinking principles and the Cocre-Ar-E (cocreate, assemble, and evaluate) framework. Phase 2 involves the technical development of the intervention using an agile scrum framework methodology, followed by usability and technical validation (alpha testing). Phase 3 consists of a pilot randomized controlled trial (n=90; 45 participants in each of the intervention and control groups) conducted in Andalusia and the Canary Islands, Spain. Outcomes include acceptability, usability, adherence, psychological well-being, and cost-effectiveness. Validated instruments include the Cancer Patients’ Stress Coping Questionnaire (CAEPO), Patient Health Questionnaire-9 (PHQ-9), Hospital Anxiety and Depression Scale (HADS), EQ-5D-5L, System Usability Scale (SUS), Client Satisfaction Questionnaire-8 (CSQ-8), Client Service Receipt Inventory (CSRI), and Mobile App Rating Scale (MARS). Quantitative analyses will include ANOVA, multilevel regression models, and cost-effectiveness analyses (incremental cost-effectiveness ratio and incremental cost-utility ratio), whereas qualitative data will be analyzed using reflexive thematic analysis supported by NVivo software.

**Results:**

Funding was awarded by the Instituto de Salud Carlos III (PI24/01193) in 2024. The SerenApp project began in January 2025 with an exploratory phase and a comprehensive literature review. The cocreation phase was conducted between September 2025 and May 2026, involving 24 breast cancer survivors and 15 multidisciplinary health care professionals. Development and alpha testing are ongoing, with recruitment expected to begin in 2027. Data analysis has not yet started, and the main study findings are expected to be published in 2028.

**Conclusions:**

This protocol describes the development and pilot evaluation of SerenApp. By integrating the perspectives of survivors, health care professionals, researchers, and technology developers, the study seeks to develop an intervention that is relevant, acceptable, and feasible within real-world health care settings. The findings generated through the cocreation process, usability evaluation, and pilot randomized controlled trial are expected to contribute to the evidence base on participatory approaches in digital mental health and inform the future refinement, implementation, and large-scale evaluation of SerenApp.

**Trial Registration:**

ClinicalTrials.gov NCT07169539; https://clinicaltrials.gov/study/NCT07169539

**International Registered Report Identifier (IRRID):**

DERR1-10.2196/87151

## Introduction

### Overview

Breast cancer is the most common malignancy among women worldwide, accounting for approximately 12.5% of all cancers [1]. Advances in early detection and treatment have substantially improved survival, with 5-year survival rates exceeding 85% in Europe and other developed regions [2,3]. However, surviving cancer does not imply being free from sequelae, as cancer survivorship is often associated with significant emotional distress. Many patients experience anxiety, depression, and stress symptoms that may persist for years after completing treatment [4-6]. It is estimated that 10% to 25% of survivors present depressive symptomatology [7,8], which negatively impact their quality of life and emotional well-being [9,10].

At the end of oncological treatment, patients transition from being classified as patients to being considered survivors, which entails profound emotional and structural changes in their health care experience. Reduced contact with health care professionals, the loss of the oncological “safety net,” and fear of recurrence often generate heightened stress, uncertainty, and feelings of abandonment [7,11,12]. These conditions may trigger or perpetuate psychological disturbances that, if not detected or treated early, interfere with daily life and hinder recovery. Consequently, the high prevalence of psychological distress in the posttreatment phase highlights the need for targeted support during survivorship and underlines the importance of early detection and intervention [7,11,13].

Although psychological therapy has proven effective in this population [14], its implementation in routine clinical practice remains limited. After discharge from oncology, patients are usually referred to primary care, where treatment for depressive and anxious symptoms continues to rely largely on pharmacological approaches despite their limitations and high relapse rates [15]. Barriers to accessing psychological interventions include the shortage of specialized professionals, the high cost of in-person psychotherapy, and the stigma associated with mental health problems [16].

The development of information and communication technologies has enabled the creation of digital interventions as an alternative or complement to face-to-face therapy, offering an opportunity to improve psychological care in this population. Digital health interventions have shown effectiveness and cost-effectiveness in reducing stress, anxiety, and depressive symptoms in women with breast cancer [17-21], serving as therapeutic adjuncts, promoting self-care and patient empowerment, and overcoming many of the limitations of conventional treatment.

Despite the growing number of mobile health apps available for people affected by breast cancer, important limitations remain. Recent reviews have shown that most commercially available apps focus primarily on breast self-examination, symptom monitoring, treatment information, or healthy lifestyle recommendations, whereas few specifically address the psychosocial and emotional needs of women during survivorship [22,23]. Furthermore, many interventions evaluated in clinical studies are not publicly available through app stores, creating a gap between research evidence and accessible resources for patients [20].

In Spain, the situation appears even more limited. A review of commercially available Spanish-language mobile apps for individuals with breast cancer identified only a limited number of apps, with overall quality ranging from moderate to low. Their content focused primarily on prevention, disease-related information, and treatment, with limited attention to emotional coping, psychological support, or survivorship care [24]. In addition, most available apps have not been developed using participatory methodologies involving patients and health care professionals, raising concerns about their relevance, usability, and long-term engagement.

Survivors of breast cancer represent a population with specific psychosocial needs that may differ from those of patients undergoing active treatment. Fear of cancer recurrence, uncertainty about the future, changes in social and family roles, and the transition from specialized oncology services to primary care require tailored support strategies. Therefore, digital interventions designed for this population should not only provide information but also deliver evidence-based psychological support, personalization, and sustained engagement while being adaptable to the health care context in which they will be implemented.

However, many existing apps show limited adherence and effectiveness, largely due to insufficient user involvement in their design [17-19,22-25]. Therefore, the design of digital health care solutions should follow a person-centered approach [26], ensuring the active participation of end users. Applying cocreation methodologies allows the development of relevant, accessible, and acceptable tools, thereby improving their utility, effectiveness, and long-term adherence [27-29]. Moreover, international organizations such as the World Health Organization (WHO) recommend that digital health solutions be designed with direct input from their end users [30]. Several conceptual frameworks have been proposed to operationalize user participation in the development of digital health interventions. Among them, design thinking has emerged as a widely adopted human-centered innovation approach that emphasizes empathy with end users, collaborative problem-solving, iterative prototyping, and continuous refinement based on user feedback [31]. This approach has been increasingly applied in health care settings to ensure that digital solutions address real-world needs and are both usable and acceptable to target populations [32,33]. Similarly, cocreation frameworks advocate the active involvement of patients and other stakeholders throughout the research and innovation process, moving beyond traditional models in which users are merely recipients of interventions [26,33-35]. In this study, the Cocre-Ar-E (cocreate, assemble, and evaluate) model [36] provides a structured participatory framework that guides the collaborative identification of needs, generation of ideas, development of prototypes, and evaluation of proposed solutions. By actively involving survivors of breast cancer, health care professionals, researchers, and technology developers, these approaches aim to enhance the relevance, acceptability, usability, and sustainability of digital health interventions in routine clinical practice [26,33,34].

Despite the promising results reported for several digital interventions targeting women with breast cancer, important challenges remain regarding survivorship-specific support, user involvement during development, personalization, and integration into health care systems.

In this context, the SerenApp project aims to develop and validate a cocreated digital intervention to support women during breast cancer survivorship. Designed for potential integration into the Spanish public health care system, SerenApp combines input from patients, health care professionals, and researchers to ensure its usability, acceptability, and clinical relevance. This paper describes the study protocol for the design, development, and pilot testing of SerenApp, addressing the current lack of detailed methodological guidance on cocreation in digital health research.

### Objectives

The overall objective of the SerenApp project is to develop and evaluate a cocreated digital psychological intervention for women in the breast cancer follow-up and survivorship phases.

Specifically, this study aims to (1) identify unmet psychosocial needs and co-design the intervention together with survivors of breast cancer and health care professionals; (2) develop and validate the usability and functionality of the digital tool; and (3) assess the feasibility, acceptability, usability, preliminary effectiveness, and cost-effectiveness of SerenApp in a pilot randomized controlled trial (RCT).

We expect that SerenApp will demonstrate acceptable feasibility, usability, and participant adherence and that the pilot trial will provide preliminary estimates of its potential effects on psychological well-being and health-related quality of life to inform a future definitive RCT.

## Methods

### Study Design

This study follows a 3-phase mixed methods design combining qualitative and quantitative approaches. The SerenApp project consists of (1) cocreation of the digital psychological intervention, (2) app development and usability validation, and (3) pilot testing of the digital tool through an RCT. This mixed methods approach allows for an in-depth understanding of user needs and experiences while ensuring that quantitative outcomes complement qualitative insights. The study adheres to the SPIRIT (Standard Protocol Items: Recommendations for Interventional Trials) guidelines (Multimedia Appendix 1 contains the SPIRIT 2025 checklist) and the Consolidated Standards of Reporting Trials of Electronic and Mobile Health Applications and Online Telehealth (CONSORT-EHEALTH) guidelines to ensure methodological transparency and reproducibility.

Figure 1 presents an overview of the study flow and timeline. The study was registered on ClinicalTrials.gov (NCT07169539) on March 31, 2025, prior to participant enrollment. The recruitment period for participants taking part in phase 1 (cocreation) began in September 2025. Participant recruitment for the cocreation and pilot-testing phases is being conducted at collaborating centers in Andalusia (Costa del Sol University Hospital, Marbella, Málaga) and the Canary Islands (Evaluation Service of the Canary Islands Health Service [SESCS], Santa Cruz de Tenerife), thereby ensuring representation from different geographic and health care contexts. Recruitment will also be supported by collaborating patient organizations, including the Spanish Association Against Cancer (AECC) in Málaga.

**Figure 1:**
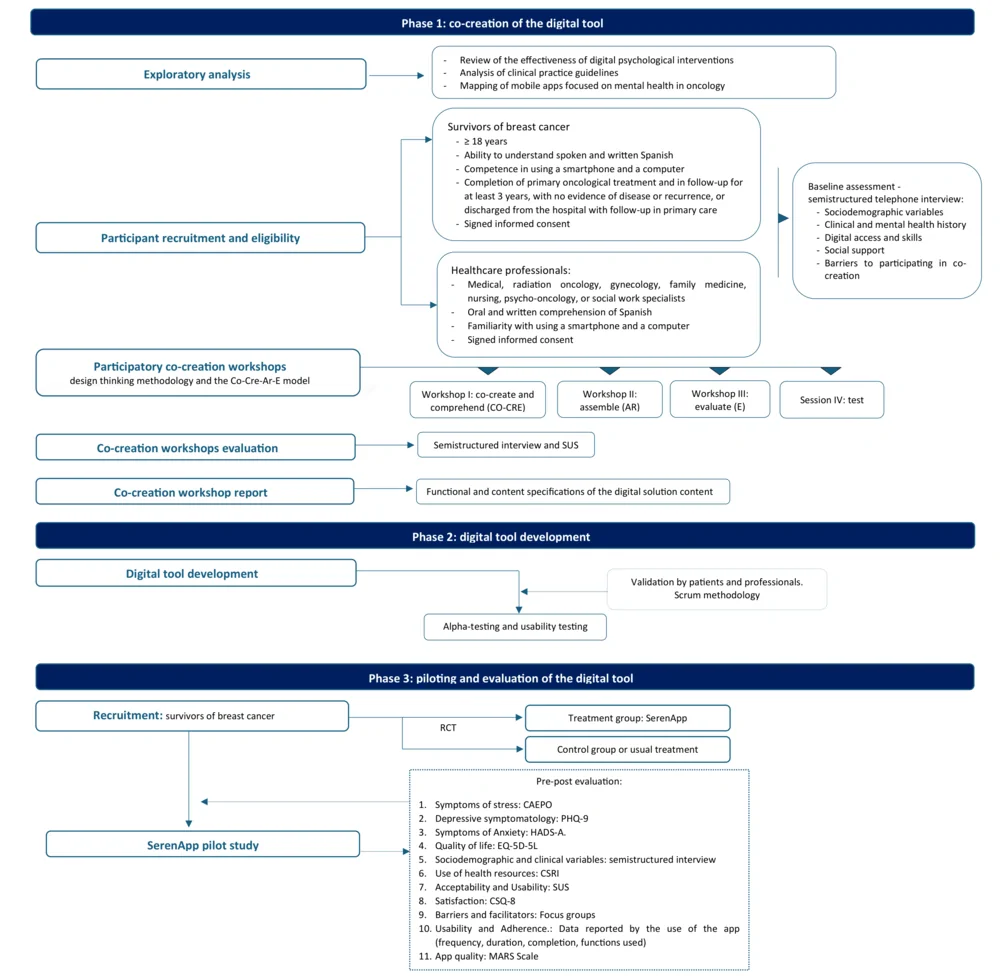
Overview of the SerenApp project design. Adapted from CONSORT (Consolidated Standards of Reporting Trials) 2010. Survivors of breast cancer underwent routine follow-up with their oncologist every 6 months. CAEPO: Cancer Patients’ Stress Coping Questionnaire; Cocre-Ar-E: cocreate, assemble, and evaluate; CSQ-8: Client Satisfaction Questionnaire-8; CSRI: Client Service Receipt Inventory; HADS-A: Hospital Anxiety and Depression Scale–Anxiety subscale; MARS: Mobile App Rating Scale; PHQ-9: Patient Health Questionnaire-9; RCT: randomized controlled trial; SUS: System Usability Scale.

### Phase 1: Cocreation of the Digital Tool

#### Overview

Phase 1 corresponds to the cocreation of the SerenApp digital intervention, the first step in the development and validation of this psychological support tool. Its primary objective is to identify the psychosocial needs, preferences, and priorities of survivors of breast cancer and to co-design the content, functionalities, and user experience of SerenApp through the active involvement of survivors and health care professionals.

#### Cocreation Methodology

This phase uses a cocreation approach that integrates principles of safety, clinical effectiveness, acceptability, and compliance with ethical, legal, and regulatory frameworks. The methodological standards established by the Spanish Network of Health Technology Assessment Agencies (RedETS) for the development and validation of digital health technologies will also be followed [37].

The process is collaborative and interdisciplinary, involving patients, health care professionals, and members of the technology development team. Iterative and structured workshops, conducted both face-to-face and online, will combine qualitative techniques typical of focus groups with participatory design methodologies, such as design thinking [31] and the Cocre-Ar-E model [36]. This methodological combination has proven useful in the development of user-centered digital health solutions [36]. Each workshop will be moderated by professionals with expertise in mental health, oncology, and cocreation. Sessions will last approximately 2 hours and will be held at 2- to 3-week intervals. Sessions will be audio- and video-recorded after obtaining informed consent, and data will be processed in accordance with the principles of confidentiality and data minimization outlined in the General Data Protection Regulation (GDPR; EU 2016/679) and Spanish Organic Law 3/2018.

Each participant will attend a total of 3 cocreation workshops and 1 final validation session, as summarized in Table 1. Workshops will include 8 to 10 participants per session to facilitate interaction and ensure diversity of perspectives.

**Table 1:** Summary of cocreation workshops.

Workshop	Objectives	Main techniques used
1. Cocreate and comprehend (COCRE)	Identify emotional needs, coping strategies, and difficulties	Empathy map, emotional traffic light, and resource suitcase
2. Assemble (AR)	Co-design content and structure of SerenApp	Lotus blossom and paper prototyping
3. Evaluate (E)	Evaluate prototype wireframes	Six thinking hats, +/–/?/idea method, and a visual design brief
4. Validation (TEST)	Test functional prototype and usability	SUS^a^ and semistructured interview

^a^SUS: System Usability Scale.

#### Exploratory Phase

Before initiating the cocreation workshops, an exploratory phase was conducted to inform the participatory design. This phase involved (1) a targeted review of the scientific literature on digital psychological interventions for survivors of breast cancer, focusing on interventions aimed at improving psychological well-being, quality of life, stress, anxiety, and depressive symptoms; (2) a review and analysis of national and international clinical practice guidelines (CPGs) related to psychological care in oncology, including those of the Spanish National Health System and the National Institute for Health and Care Excellence (NICE); and (3) a market mapping of mobile apps addressing mental health in oncology. The app mapping involved searches of the Google Play Store and Apple App Store to identify applications targeting psychological well-being, emotional support, stress management, anxiety, depression, and survivorship care in people affected by cancer. Apps were reviewed to assess their content, functionalities, level of evidence, and suitability for the Spanish clinical context. Findings from this exploratory phase guided the design of workshop materials, informed the selection of topics and activities included in the cocreation process, and ensured that SerenApp addressed identified needs and gaps in psychological support for survivors of breast cancer while incorporating evidence-based recommendations and relevant functionalities identified in existing digital tools.

#### Selection and Evaluation of Participants

##### Overview

Two separate groups will be formed, each consisting of up to 25 participants: 1 group of health care professionals and 1 group of survivors of breast cancer. This sample size was considered sufficient to ensure diversity of perspectives while maintaining manageable group dynamics during the cocreation workshops. Each participant will be assessed at 2 time points: baseline and post–cocreation evaluation.

Survivors of breast cancer will be identified through oncology follow-up consultations, collaborating clinical services, and patient associations. Health care professionals involved in participant care will provide information about the study to women who meet the inclusion criteria and will request permission for a member of the research team to contact them. Interested women will subsequently be screened for eligibility by trained researchers and invited to participate after providing written informed consent.

##### Survivors of Breast Cancer

Inclusion criteria include (1) being aged 18 years or older; (2) the ability to understand spoken and written Spanish; (3) access to a smartphone or computer and basic digital skills; (4) completion of primary oncological treatment (surgery, chemotherapy, radiotherapy, immunotherapy, or biological treatments) at least 3 years earlier, with no evidence of disease or recurrence, and currently under medical oncology follow-up (every 4 to 6 months) or discharged from hospital care but under periodic follow-up in primary care; and (5) signed informed consent.

Exclusion criteria include (1) the presence of moderate-to-severe depressive symptomatology (score ≥19 on the Patient Health Questionnaire-9 [PHQ-9]); (2) the presence of moderate-to-severe anxiety symptoms (score ≥11 on the Hospital Anxiety and Depression Scale–Anxiety subscale [HADS-A]); and (3) a diagnosis of a severe mental disorder, cognitive impairment, or another condition interfering with active participation in the cocreation process, documented in the clinical record.

Participants receiving psychological or psychopharmacological treatment at the time of the study will not be excluded, provided that they do not meet the other exclusion criteria.

The baseline evaluation includes a semistructured interview confirming eligibility and collecting sociodemographic, oncological, mental health, and digital literacy data, perceived social support, barriers, and expectations regarding SerenApp.

The final evaluation includes a semistructured interview assessing the overall experience with the participatory process, perceived usefulness, adequacy, design, accessibility, and therapeutic potential of the app; integration of participant input; and barriers encountered. The System Usability Scale (SUS) [38] will also be administered to assess perceived usability.

##### Health Care Professionals

Health care professionals participating in the cocreation process will be recruited using purposive sampling to ensure representation from the different disciplines involved in breast cancer care. Potential participants will be identified through collaborating institutions and invited directly by members of the research team.

Inclusion criteria include (1) health care professionals involved in the care of survivors of breast cancer, (2) fluency in Spanish, (3) competence in using smartphones and computers; and (4) provision of written informed consent.

Exclusion criteria include significant difficulty using digital tools.

The baseline evaluation includes an individual semistructured interview collecting sociodemographic and professional data, prior mental health care, digital health experience, digital skills, and expectations regarding SerenApp.

The final evaluation includes a semistructured interview exploring the cocreation experience, perceived usefulness and clinical applicability, evaluation of the app’s design and functionalities, changes in attitudes toward digital tools, and barriers and facilitators. The SUS will again be used to assess perceived usability.

#### Evaluation of the Cocreation Process

Data from the workshops (attendance, transcripts, and satisfaction) and post-cocreation interviews will be analyzed using a mixed methods approach [39]. Qualitative data will be analyzed thematically using NVivo (Lumivero) software, with codes derived both inductively and deductively from the research questions. Quantitative data from the SUS will be summarized descriptively (mean, SD, and percentage of participants scoring ≥68). Findings from this evaluation will inform refinements to SerenApp’s design, content, and usability before proceeding to the pilot-testing phase. Qualitative rigor will be ensured through triangulation across participant groups, double coding of a subset of transcripts, and iterative peer review within the research team.

#### Cocreation Workshop Report

After completing and evaluating the cocreation process and integrating the data from the workshops with the evidence gathered during the exploratory phase (literature review, CPG analysis, and market mapping), a comprehensive technical report will be developed. The report will detail the functional and content specifications, validated modules, technical and design requirements, and provide recommendations for further development, clinical implementation, and evaluation. It will serve as a reference framework for both the technology development and research teams in the subsequent phases of the SerenApp project.

### Phase 2: Digital Tool Development

#### Overview

This phase focuses on the design, development, and validation of the SerenApp digital intervention. Building on the insights and specifications obtained during the cocreation phase, the goal is to translate those results into a functional, user-centered, and clinically relevant tool tailored to the real needs of survivors of breast cancer. We will also verify that the tool works correctly and is ready for pilot evaluation. The development will be carried out in close collaboration with a specialized technology company with expertise in health technologies.

#### Digital Tool Development Methodology

The digital intervention will be developed following the agile scrum framework methodology, which enables iterative and progressive creation through short development cycles (sprints) and frequent reviews. This approach facilitates continuous feedback from the research team, patients, and health care professionals involved in the cocreation phase.

Close collaboration will be maintained between the multidisciplinary research team and the technology company to ensure proper integration of clinical content into the app’s interface and functionalities. This coordination will guarantee that the tool meets both user needs and usability and accessibility criteria. Monthly online meetings will be held to review progress, validate design decisions, and introduce adjustments in each iteration.

#### Regulatory Compliance and Data Security

Development will comply with current data protection legislation, including Spanish Organic Law 3/2018 and the GDPR (EU 2016/679). Ethical, deontological, and legal principles applicable to digital health design will also be followed. To guarantee confidentiality, integrity, and security, advanced encryption mechanisms and secure storage protocols will be implemented for all sensitive data managed by the tool. Periodic security audits will be conducted throughout the development process to detect and address potential vulnerabilities before the pilot phase.

#### Alpha Testing and Usability Evaluation

Before proceeding to the pilot phase (beta testing), an alpha-testing phase will be conducted to evaluate usability and user experience. This phase will ensure that the app accurately reflects the defined therapeutic content and modules and meets functional, accessibility, and usability standards.

Alpha testing will be conducted in the following two stages: (1) internal testing, performed by the research team to validate basic functionality, navigation, and technical performance; and (2) controlled-environment testing, conducted with patients and professionals from the cocreation process to evaluate navigation, accessibility, and interaction with core features (eg, therapeutic tools, support resources, or psychological assessments).

Usability will be assessed through defined tasks and evaluated using the SUS and qualitative interaction logs. Testing sessions will include (1) task-based interaction with prototypes; (2) recording of times, errors, and satisfaction levels; and (3) collection of qualitative feedback for iterative refinement.

Findings will be integrated into subsequent development sprints to ensure adherence to accessibility, usability, and user engagement criteria. Continuous feedback from the research team and cocreation participants will guide iterative improvements until the tool is validated for pilot testing.

### Phase 3: Piloting and Evaluation of the Digital Tool

#### Study Design

After developing the SerenApp tool, the third phase will evaluate its feasibility, preliminary effectiveness, and cost-effectiveness through a pilot RCT with 2 parallel groups (intervention vs usual care) [40-42]. The objective of this phase is to assess the preliminary impact of SerenApp compared to usual care, examining its effectiveness and cost-effectiveness, as well as its acceptability, usability, and feasibility in real clinical settings.

Patient recruitment procedures and inclusion and exclusion criteria will be identical to those applied in the cocreation phase, ensuring sample comparability across study phases. Additionally, qualitative data on the app’s acceptability and usability will be collected through focus groups to identify potential areas for improvement before large-scale implementation.

#### Methodology of the Pilot Study

##### Sample Size

The sample size calculation will be based on the ability to detect differences between the pilot app and usual care, assuming a difference in the primary variable of at least 0.5 SDs and a statistical power of 80%. The effect size was obtained from previous studies [43-49] that examined eHealth psychological interventions vs usual care, considering that the expected dropout rate for this type of study ranges from 15% to 30% and that pilot studies typically require approximately 30% fewer patients [50,51]. Therefore, a total sample size of 90 participants is estimated.

##### Participants and Procedure

Participants will be recruited for the pilot study, comprising 45 women assigned to the intervention group (testing the digital tool) and 45 assigned to the control group (usual care). Recruitment will take place through oncology follow-up consultations, collaborating clinical services, and patient associations at the participating centers. Potential participants will receive verbal and written information about the study during routine follow-up visits. Women expressing interest in participation will be contacted by a member of the research team to verify eligibility and complete the informed consent process. Following provision of informed consent, an evaluating researcher (ER), independent of the intervention team, will conduct a baseline assessment to confirm eligibility, collect sociodemographic and clinical information, and administer the psychological outcome measures. Based on this assessment, the ER will determine participants’ suitability for inclusion in the study. Randomization will be performed using a computer-generated random allocation sequence. Outcome assessments will be conducted by the ER, independent of the intervention team, throughout the study.

Participants in the intervention group will receive a link to access SerenApp, complete baseline and posttreatment assessments, and use the tool for 8 weeks. Those in the control group will receive a link to complete preintervention and postintervention evaluations only after the same 8-week period. In parallel, the ER will extract relevant clinical data from participants’ electronic medical records.

Upon completion of the pilot phase, participants in the intervention group will be invited to participate in focus groups (8 to 10 women per group, lasting approximately 60 to 90 minutes) to provide feedback on usability, content adequacy, accessibility, and perceived barriers and facilitators. This feedback will be used to refine and optimize the SerenApp intervention prior to large-scale evaluation. Finally, quantitative and economic analyses will be conducted to assess the intervention’s preliminary effectiveness and cost-effectiveness.

#### Outcome Measures

Primary and secondary outcomes will be assessed at baseline and postintervention using validated psychometric instruments, usability measures, and health economic indicators. The primary outcomes of the pilot study are (1) feasibility of the intervention, assessed through recruitment, retention, completion, and adherence rates; (2) acceptability and usability of SerenApp, assessed using the SUS, participant satisfaction measures, and qualitative feedback; and (3) preliminary effectiveness on psychological well-being, including stress, anxiety, and depressive symptoms.

Progression to a future definitive RCT will be guided by predefined feasibility criteria, in accordance with recommendations for pilot and feasibility studies [52-54]. Specifically, the study will be considered feasible if at least 70% of the planned sample is recruited within the recruitment period, participant retention is ≥80%, completion of outcome assessments is ≥80%, and adherence to the intervention (completion of the core intervention modules) is ≥70%. These quantitative criteria will be interpreted alongside qualitative findings on acceptability and usability to determine whether further refinement of the intervention is required before proceeding to a fully powered RCT.

The secondary outcomes are (1) health-related quality of life, (2) adherence and engagement with the intervention, (3) user satisfaction, (4) health care resource use, and (5) cost-effectiveness and cost-utility outcomes.

#### Instruments

The following instruments will be used to assess these outcomes:

#### Cancer Patients’ Stress Coping Questionnaire

The Cancer Patients’ Stress Coping Questionnaire (CAEPO) [55], developed specifically for oncology patients in the Spanish population, will be used to assess stress symptoms. The CAEPO measures the cognitive and behavioral coping strategies that oncology patients use to cope with their illness. It consists of 40 items rated on a 4-point Likert scale (scores 0-3) across 7 subscales: confrontation and active fighting, self-control and emotional control, seeking social support, anxiety and anxious worry, passivity and passive resignation, escape and distancing, and denial.

#### PHQ-9

The PHQ-9 [56,57] is a brief, 9-item self-report measure designed to screen for a major depressive episode in the last 2 weeks, following the major depression criteria of the *DSM-IV* (*Diagnostic and Statistical Manual of Mental Disorders, Fourth Edition*), as well as to assess its severity. Responses are rated on a Likert scale from 0 (not at all) to 3 (nearly every day). The total score suggests different levels of depression. Scores range from 0 to 27. In general, a total score of 10 or more suggests the presence of depression: no depression (score 0-4), mild depression (score 5-9), moderate depression (score 10-14), moderately severe depression (score 15-19), and severe depression (score 20-27).

#### Hospital Anxiety and Depression Scale

The Hospital Anxiety and Depression Scale (HADS) [58,59] is a 14-item self-administered questionnaire composed of two 7-item subscales, each on a 0-3 Likert scale. The HADS-A refers to the odd-numbered items, and the Hospital Anxiety and Depression Scale–Depression subscale (HADS-D) to the even-numbered items, with a score range for each subscale of 0-21. For each subscale, the score is obtained by summing the scores of its items. The interpretation criteria are as follows: normal (score 0-7), possible case of anxiety or depression (score 8-10), and case of anxiety or depression (score 11-21).

#### EQ-5D-5L

The EQ-5D-5L [60] is a generic instrument for measuring health-related quality of life in which individuals first rate their health status based on severity levels for each dimension (descriptive system) and then on a more general visual analog scale. The descriptive system contains 5 health dimensions (mobility, self-care, usual activities, pain and discomfort, and anxiety and depression), each with 5 severity levels coded from 1 to 5 [61].

#### Client Service Receipt Inventory

The Client Service Receipt Inventory (CSRI) [62] is one of the most widely used tools for measuring resource use. It describes and measures service use patterns as a basis for estimating associated costs in the areas of health care, social care, and community care. In the present study, the Spanish version will be used to collect retrospective data (last 2 months) on medication and service use.

#### SUS

To evaluate the app’s acceptability and usability, the SUS [38] will be used. It consists of 10 items with responses ranging from 1 (strongly disagree) to 5 (strongly agree). It provides a global usability score ranging from 0 to 100, where a score greater than 68 indicates good usability. It is widely used in the evaluation of software and mobile health apps.

#### Client Satisfaction Questionnaire-8

The Client Satisfaction Questionnaire-8 (CSQ-8) [63] is an 8-item questionnaire designed to measure user satisfaction with services received. Developed as a Likert scale (scores 1-4), it evaluates aspects such as the quality of the service, its adequacy for user needs, and the likelihood of recommending it to others.

#### Sociodemographic and Clinical Variables

Sociodemographic data (age, autonomous community of residence, education level, income level, marital status, living arrangement, number of children, dependents, and employment status) will be collected through a semistructured interview. Clinical data (Charlson Comorbidity Index; tumor, node, metastasis [TNM] stage; and types of treatment up to hospital discharge) will be collected from the patient’s electronic medical record.

#### Intervention Adherence and Engagement

We will collect data on frequency of use (number of weekly accesses), average session time (duration in minutes of each session), completion rate (percentage of users who complete the intervention modules), and engagement (use of features such as reminders and wellness tracking).

#### Barriers and Facilitators

Participants’ experiences and perceptions after using the digital solution will be discussed through semistructured interviews. This methodology will allow us to identify barriers in its adoption, usability, and effectiveness, as well as factors that promote its use and acceptance. Analysis of these interviews will provide key insights to improve the app and optimize its impact on users.

#### Mobile App Rating Scale

The Mobile App Rating Scale (MARS) [64] evaluates the quality of health apps across 4 domains: engagement, functionality, aesthetics, and information.

### Data Analysis

A mixed methods approach will be applied, integrating quantitative, qualitative, and economic analyses to provide a comprehensive evaluation of the SerenApp pilot. A qualitative analysis of the focus group data will be conducted, along with quantitative analyses assessing the effectiveness, reliability, and validity of the digital psychological intervention, as well as cost-effectiveness and cost-utility analyses.

### Effectiveness Analysis

Given the pilot nature of this study, all quantitative analyses will be considered exploratory and will primarily aim to estimate the direction and magnitude of intervention effects rather than formally test hypotheses. Descriptive statistics, effect size estimates, and 95% CIs will be emphasized to inform the design and sample size calculation of a future definitive RCT. Between-group comparisons will therefore be interpreted cautiously.

Exploratory between-group comparisons of all outcome variables measured at baseline and postintervention will be conducted using repeated-measures ANOVA for normally distributed variables or appropriate nonparametric tests when distributional assumptions are not met. More complex multivariable analyses, including multilevel regression models, will be performed when appropriate to account for repeated measures and potential confounding factors. The magnitude of intervention effects will be quantified using effect size estimates and the number needed to treat, where appropriate. All analyses will be performed using SPSS (version 28; IBM Corp).

### Effectiveness, Cost-Effectiveness, and Cost-Utility Analysis

A complete economic evaluation will be performed to assess the costs and health outcomes of using the app compared to usual care during the study period, following international recommendations [65]. This analysis will integrate both cost-effectiveness and cost-utility components. For the cost-effectiveness analysis, the primary outcome measures will be differences in CAEPO, PHQ-9, and HADS scores. For the cost-utility analysis, the main outcome will be the incremental cost per quality-adjusted life-year gained, derived from EQ-5D-5L results using the algorithm of Ramos-Goñi et al [66]. Analyses will adopt the National Health System perspective, including only direct health care costs. Costs will be estimated at the patient level, combining resource use with corresponding unit costs obtained from national sources [67]. Uncertainty will be assessed using both parametric and nonparametric methods, including bootstrapping. Incremental cost-effectiveness ratios and incremental cost-utility ratios will be computed with confidence intervals, and cost-effectiveness acceptability curves will be generated. Deterministic sensitivity analyses will be performed to evaluate the robustness of the results.

### Qualitative Analysis

Qualitative data derived from cocreation workshops, semistructured interviews, and focus groups will be analyzed using reflexive thematic analysis following the recommendations of Braun and Clarke [68]. Audio recordings will be transcribed verbatim, anonymized, and imported into NVivo software to facilitate data management and coding. The analysis will follow six iterative phases: (1) familiarization with the data through repeated reading of transcripts, (2) generation of initial codes, (3) construction of preliminary themes, (4) review and refinement of themes in relation to the coded extracts and the entire dataset, (5) definition and naming of themes, and (6) production of the final analytical report. Both deductive and inductive approaches will be used, allowing the identification of themes informed by the study objectives while remaining open to unexpected insights emerging from participants’ experiences.

Reflexivity will be promoted throughout the research process through regular discussions among members of the multidisciplinary research team regarding assumptions, interpretations, and potential sources of bias. Analytical decisions, coding processes, and theme development will be documented through an audit trail to ensure transparency and methodological rigor.

To enhance trustworthiness, several strategies will be implemented. Credibility will be supported through triangulation across participant groups (survivors of breast cancer and health care professionals) and investigator discussions during data interpretation. Dependability and confirmability will be strengthened through systematic documentation of coding decisions and peer debriefing within the research team. Transferability will be facilitated through detailed descriptions of the study context, participant characteristics, and cocreation procedures, enabling readers to assess the applicability of the findings to other settings.

Qualitative findings will be integrated with quantitative results to provide a comprehensive understanding of participants’ experiences, usability perceptions, barriers, facilitators, and recommendations for improving the SerenApp intervention.

### Ethical Considerations

The study will adhere to Good Clinical Practice (GCP) standards and the ethical principles of the Declaration of Helsinki and its subsequent revisions. Ethical approval was obtained from the Research Ethics Committee of the Costa del Sol University Hospital (reference number 002_nov23-PI2-APP Depresión y CaMama). Clinical data will be stored separately from personal identifiers, and all databases will be anonymized and encrypted, with access restricted to authorized study staff. All data handling will comply with Spanish Organic Law 3/2018 on Data Protection and the GDPR (EU 2016/679). The technology partner developing SerenApp will sign a confidentiality and good practice agreement regarding participant data management. All participants will receive written and verbal information about the study objectives, procedures, and data management and will provide written informed consent prior to participation. All audiovisual materials obtained during cocreation sessions will be securely deleted upon study completion. Research staff will maintain strict confidentiality and comply with ethical and legal data protection standards.

Results will be disseminated through peer-reviewed journals, national and international conferences, and collaborations with health care institutions to support the potential integration of SerenApp into the public health system. Summarized results will also be shared with participants and relevant stakeholders. Deidentified participant data and statistical code will be available upon reasonable request to the corresponding author after publication, in accordance with applicable data protection regulations.

## Results

The SerenApp project was funded by the Instituto de Salud Carlos III (ISCIII) under grant PI24/01193 in 2024. The study commenced in January 2025 with preparatory activities, including a review of the scientific literature on digital psychological interventions for survivors of breast cancer, an analysis of relevant CPGs, a mapping of currently available mobile health apps, and registration of the study on ClinicalTrials.gov (NCT07169539). Table 2 summarizes the anticipated timeline of the SerenApp project. Recruitment for the cocreation phase (Phase 1) began in September 2025. A total of 24 survivors of breast cancer and 15 health care professionals participated in the cocreation process. Health care professionals represented a multidisciplinary team, including specialists in medical oncology, radiation oncology, integrative oncology, breast surgery, gynecology, nursing, primary care, psychology, and social work.

**Table 2:** Timeline for the SerenApp project.

Phase and activity			Timeline
**Phase 1**			
	Project funding (ISCIII^a^ grant number PI24/01193)	2024	
	Preparatory phase: literature review, CPG^b^ review, app mapping, and protocol development	January-August 2025	
	ClinicalTrials.gov registration	March 2025	
	Recruitment for cocreation participants	September 2025	
	Cocreation workshops	October 2025-May 2026	
**Phase 2**			
	App development and alpha testing	June 2026-February 2027	
**Phase 3**			
	Pilot RCT^c^ recruitment	March-April 2027	
	Intervention and follow-up assessments	2027	
	Data analysis	End of 2027	
	Dissemination of findings	2028	

^a^ISCIII: Instituto de Salud Carlos III.

^b^CPG: clinical practice guideline.

^c^RCT: randomized controlled trial.

Cocreation workshops were conducted between October 2025 and May 2026 to identify users’ unmet psychosocial needs and collaboratively define the content, functionalities, and design requirements of SerenApp. Findings from the workshops informed the development of the intervention structure and the creation of the first wireframes and functional prototypes of the app.

Phase 2, consisting of the technical development and usability validation of SerenApp, will take place between June 2026 and February 2027. This phase includes software development, iterative refinement processes, and alpha testing to assess usability, functionality, accessibility, and user experience. Recruitment for the pilot RCT (phase 3) is expected to begin in March-April 2027 following completion of the development and validation process. A total of 90 survivors of breast cancer will be enrolled and randomly allocated to either the SerenApp intervention group or the usual care control group. Data collection and quantitative, qualitative, and economic analyses are expected to be completed by the end of 2027. Findings regarding feasibility, acceptability, usability, adherence, preliminary effectiveness, and cost-effectiveness are expected to be disseminated in 2028 and will inform the design of a future large-scale RCT.

## Discussion

### Principal Findings

This study presents a research protocol for developing a digital mental health intervention using a cocreation and person-centered approach. This design integrates patients, health care professionals, and digital design experts with the goal of improving access to psychological care for women with breast cancer and promoting their well-being and quality of life beyond the oncology treatment period. This approach is key to ensuring that the digital solution is both useful and contextually relevant, addressing one of the main limitations of previous digital interventions: limited adherence and lack of personalization [69]. Traditionally, patient involvement in research has been one-directional, with limited opportunities for participants to contribute to intervention design or implementation. However, recent literature emphasizes the importance of conducting research in partnership with stakeholders, including patients, clinicians, and developers. A user-centered innovation model can accelerate the development process and enhance effectiveness by aligning solutions with users’ real needs [70,71].

### Comparison With Prior Work

Compared with previously published digital interventions for women with breast cancer, SerenApp shares the objective of improving psychological well-being and supporting self-management but differs in several important aspects. Existing interventions, such as the Breast Cancer e-Support Program developed by Zhu et al [72], iCanThrive [73], and StressProffen [74], have demonstrated promising effects on psychological outcomes, quality of life, coping skills, and symptom management among women affected by cancer. However, many of these interventions were developed for patients undergoing active treatment, targeted heterogeneous cancer populations, or provided limited information regarding the extent of patient involvement in the design and development process. Furthermore, a recent systematic review and meta-analysis [75] highlighted the potential of digital interventions to improve psychosocial outcomes and quality of life among women with breast cancer while also identifying substantial heterogeneity in intervention characteristics, outcome measures, and implementation approaches. These findings suggest that further research is needed to develop more tailored, user-centered, and survivorship-focused interventions.

One of the most persistent challenges reported in the digital mental health literature is maintaining user engagement and adherence over time. Despite demonstrated effectiveness, many interventions experience high attrition rates and reduced use after the initial stages of participation. Perski et al [76] proposed that engagement is influenced not only by the usability of a digital intervention but also by its perceived relevance, personalization, and ability to meet users’ expectations and needs. Similarly, the person-based approach described by Yardley et al [77] emphasizes that interventions are more likely to be accepted and used when they are grounded in a deep understanding of users’ experiences, preferences, and contextual realities. These findings are particularly relevant in cancer survivorship, where emotional needs, fear of recurrence, and adaptation challenges may evolve over time and require sustained support.

In response to these limitations, SerenApp was conceived as a cocreated digital intervention that actively involves survivors of breast cancer, health care professionals, researchers, and technology developers throughout the design process. This approach is consistent with growing evidence suggesting that participatory methodologies can improve the relevance, usability, acceptability, and long-term engagement of digital health interventions [33,77]. Moreover, unlike many commercially available breast cancer apps, which primarily focus on information provision, symptom monitoring, or lifestyle recommendations, SerenApp specifically targets psychosocial well-being during survivorship and incorporates a structured evaluation of usability, feasibility, preliminary effectiveness, and cost-effectiveness. By combining evidence-based psychological content with a cocreation framework and adaptation to the Spanish health care context, the present study aims to address several of the evidence gaps identified in previous research and contribute to the methodological literature on the development of person-centered digital interventions for cancer survivorship.

Over the last decade, digital mental health interventions have grown exponentially, including mobile apps, social media–based tools, chatbots, and virtual reality [78]. This trend accelerated during the COVID-19 pandemic, which spurred unprecedented technological advances [79,80] and expanded the potential of digital tools to improve access to psychological care. Despite this promise, these interventions still face significant challenges regarding user engagement and long-term adoption. The absence of user perspectives during development has been identified as a key determinant of poor adherence. Although human-centered design approaches have attempted to address this issue, their application in mental health remains limited and often lacks genuine end user participation during the development process.

For the development of SerenApp, it was considered essential to integrate the needs, expectations, and concerns of women who have experienced breast cancer from the earliest design stages. We also incorporated the perspectives of health care professionals to identify gaps in psychosocial care. An initial prototype will be tested before large-scale validation, allowing for iterative adjustments to optimize functionality and clinical utility. We expect that the results of the SerenApp pilot will underscore the importance of cocreation in developing digital tools for psychological support among survivors of breast cancer. Evaluating barriers and facilitators through focus groups will help identify key aspects of usability, acceptability, and adherence. Moreover, following the cost-effectiveness analysis, if SerenApp is found to be feasible, acceptable, and effective, it could represent a viable and sustainable complement to face-to-face psychological interventions, particularly in settings with limited mental health resources.

In future phases, the project aims to expand its evaluation with larger-scale, long-term studies to consolidate evidence on its impact on users’ quality of life and to support its integration into public health care systems.

### Strengths and Limitations

This study has several strengths. First, it adopts a cocreation approach that actively involves survivors of breast cancer and health care professionals throughout the development process, increasing the likelihood that the resulting intervention will be relevant, acceptable, and responsive to users’ needs. Second, the mixed methods design combines qualitative and quantitative approaches, allowing a comprehensive evaluation of usability, feasibility, acceptability, preliminary effectiveness, and cost-effectiveness. Third, the inclusion of participants from different health care settings and geographic regions may enhance the relevance and transferability of the findings within the Spanish health care context.

Several limitations should also be acknowledged. As a pilot study, the sample size is not powered to establish definitive conclusions regarding effectiveness. In addition, participants will be recruited from specific health care settings in Spain, which may limit the generalizability of the findings to other populations or health care systems. The study may also be subject to selection bias, as participants willing to engage in digital health interventions may have greater motivation or digital literacy than the broader population of survivors of breast cancer. Nevertheless, the pilot design is intended to identify implementation challenges, refine the intervention, and generate data to inform future large-scale evaluations. Accordingly, any between-group differences observed will be interpreted as preliminary estimates of potential intervention effects rather than confirmatory evidence of effectiveness. The primary purpose of this pilot trial is to evaluate feasibility, acceptability, usability, recruitment, and retention processes and to generate parameters that will inform the design of a fully powered RCT.

### Dissemination and Future Directions

The findings from the cocreation process, usability evaluation, and pilot trial will be disseminated through peer-reviewed publications, conference presentations, and knowledge-transfer activities involving health care professionals, patient organizations, and decision-makers. The results will also inform the future development of SerenApp and support the design of a fully powered RCT. Future research should evaluate the long-term effectiveness, implementation, and scalability of SerenApp in larger and more diverse populations. If proven feasible and acceptable, the intervention could contribute to improving access to evidence-based psychological support for survivors of breast cancer within routine health care services.

### Conclusions

This protocol describes the development and evaluation of SerenApp, a cocreated digital psychological intervention designed to support survivors of breast cancer during follow-up and survivorship. By integrating the perspectives of survivors, health care professionals, researchers, and technology developers, the study seeks to develop an intervention that is relevant, acceptable, and feasible within real-world health care settings. The findings generated through the cocreation process, usability evaluation, and pilot RCT are expected to contribute to the evidence base on participatory approaches in digital mental health and inform the future refinement, implementation, and large-scale evaluation of SerenApp.

## Data Availability

Data sharing is not applicable to this article as no datasets were generated or analyzed during this study.

## Appendix

Multimedia Appendix 1 SPIRIT 2025 checklist.

## Abbreviations

AECC: Spanish Association Against Cancer
CAEPO: Cancer Patients’ Stress Coping Questionnaire
Cocre-Ar-E: cocreate, assemble, and evaluate
CONSORT-EHEALTH: Consolidated Standards of Reporting Trials of Electronic and Mobile Health Applications and Online Telehealth
CPG: clinical practice guideline
CSQ-8: Client Satisfaction Questionnaire-8
CSRI: Client Service Receipt Inventory
DSM-IV: Diagnostic and Statistical Manual of Mental Disorders, Fourth Edition
ER: evaluating researcher
GCP: Good Clinical Practice
GDPR: General Data Protection Regulation
HADS: Hospital Anxiety and Depression Scale
HADS-A: Hospital Anxiety and Depression Scale–Anxiety subscale
HADS-D: Hospital Anxiety and Depression Scale–Depression subscale
ISCIII: Instituto de Salud Carlos III
MARS: Mobile App Rating Scale
NICE: National Institute for Health and Care Excellence
PHQ-9: Patient Health Questionnaire-9
RCT: randomized controlled trial
RedETS: Spanish Network of Health Technology Assessment Agencies
SESCS: Evaluation Service of the Canary Islands Health Service
SPIRIT: Standard Protocol Items: Recommendations for Interventional Trials
SUS: System Usability Scale
TNM: tumor, node, metastasis
WHO: World Health Organization
