# Supplementary material for: Cocreation of a Digital Psychological Intervention to Improve Mental Health Among Survivors of Breast Cancer (SerenApp): Protocol for a Usability and Pilot Randomized Controlled Trial
Source: JMIR Res Protoc. 2026 Sep 30;15:e87151. doi: 10.2196/87151 (PMC13669711; doi:10.2196/87151)
Supplement: Supplementary file 1 [file resprot_v15i1e87151_app1.pdf]

SPIRIT 2025 checklist of items to address in a randomized trial protocol*

Section / Topic	No	SPIRIT 2025 checklist item description	Reported on page no.	
**Administrative information**				
Title and structured summary	1a	Title stating the trial design, population, and interventions, with identification as a protocol	Title page (p. 1)	The title specifies the study design, population (breast cancer survivors), and intervention (SerenApp digital psychological tool).
	1b	Structured summary of trial design and methods, including items from the World Health Organization Trial Registration Data Set	Abstract (p. 2)	The abstract provides a structured summary including background, objectives, design, methods, and expected outcomes.
Protocol version	2	Version date and identifier	Title page (p. 1)	Version 3: October 20, 2025.
Roles and responsibilities	3a	Names, affiliations, and roles of protocol contributors	Title page (p. 1)	Includes main investigators, institutional affiliations, and contributor roles.
	3b	Name and contact information for the trial sponsor	Title page (p. 1)	Sponsor: Instituto de Salud Carlos III (ISCIII), Project Code PI24/01193. Contact details provided.
	3c	Role of trial sponsor and funders in design, conduct, analysis, and reporting of trial; including any authority over these activities	Acknowledgements and Funding (p. 21)	The funder had no role in study design, data collection, analysis, interpretation, or publication decisions.
	3d	Composition, roles, and responsibilities of the coordinating site, steering committee, endpoint adjudication committee, data management team, and other individuals or groups overseeing the trial, if applicable	Not applicable	No formal steering or adjudication committees were established due to the exploratory pilot design.
**Open science**				
Trial registration	4	Name of trial registry, identifying number (with URL), and date of registration. If not yet registered, name of intended registry	Methods – Study design and setting (p. 5)	Registered on ClinicalTrials.gov (NCT07169539) on March 31, 2026, prior to participant enrolment.
Protocol and statistical analysis plan	5	Where the trial protocol and statistical analysis plan can be accessed	Methods – Phases 1–3 (pp. 5–18)	The full protocol and statistical analysis plan are detailed within this document. Supplementary materials (e.g., SPIRIT checklist) provided for transparency.
Data sharing	6	Where and how the individual de-identified participant data (including data dictionary), statistical code, and any other materials will be accessible	Ethics and dissemination (pp. 18–19)	De-identified participant data and statistical code will be available upon reasonable request to the corresponding author, in compliance with GDPR and Spanish data protection law.
Funding and conflicts of interest	7a	Sources of funding and other support (e.g., supply of drugs)	Funding (p. 21)	Funded by Instituto de Salud Carlos III (PI24/01193), co-financed by the European Union (ERDF).

	7b	Financial and other conflicts of interest for principal investigators and steering committee members	Conflicts of Interest (p. 20)	The authors declare no financial or competing interests.
Dissemination policy	8	Plans to communicate trial results to participants, healthcare professionals, the public, and other relevant groups (e.g., reporting in trial registry, plain language summary, publication)	Ethics and dissemination (pp. 18–19)	Findings will be disseminated through peer-reviewed publications, conferences, collaborations with healthcare institutions, and summaries for participants.
**Introduction**				
Background and rationale	9a	Scientific background and rationale, including summary of relevant studies (published and unpublished) examining benefits and harms for each intervention	Introduction (pp. 3–5)	Reviews evidence on psychological distress among breast cancer survivors and the effectiveness of digital interventions.
	9b	Explanation for choice of comparator	Methods – Phase 3: Piloting and Evaluation (p. 12)	The control group receives usual care, representing the standard follow-up in oncology.
Objectives	10	Specific objectives related to benefits and harms	Introduction, pp.4-5	Main objective: to develop and evaluate SerenApp, a co-created digital intervention to reduce stress, anxiety, and depression. Secondary objectives: assess usability, acceptability, and cost-effectiveness.
**Methods: Patient and public involvement, trial design**				
Patient and public involvement	11	Details of, or plans for, patient or public involvement in the design, conduct, and reporting of the trial	Methods - Phases 1-3 (pp. 6 -13).	In SerenApp, patients, healthcare professionals, and design experts actively participate throughout all phases. The co-creation process integrates users in the design, development, and pilot evaluation of the app, following a person-centered, participatory methodology.
Trial design	12	Description of trial design including type of trial (e.g., parallel group, crossover), allocation ratio, and framework (e.g., superiority, equivalence, non-inferiority, exploratory)	Methods - Phase 3: Piloting and Evaluation (pp. 12 -18).	Randomized controlled pilot study with two parallel groups (intervention vs. usual care), 1:1 allocation ratio. The trial follows an exploratory framework to assess feasibility, usability, and preliminary effectiveness.
**Methods: Participants, interventions, and outcomes**				
Trial setting	13	Settings (e.g., community, hospital) and locations (e.g., countries, sites) where the trial will be conducted	Methods – Phase 1: Co-creation (p. 7) and Phase 3: Piloting and Evaluation (p. 12)	Conducted in two Spanish public healthcare centers: Hospital Universitario Costa del Sol (Marbella, Andalusia) and the Evaluation Service of the Canary Islands Health Service (Santa Cruz de Tenerife).
Eligibility criteria	14a	Eligibility criteria for participants	Methods – Phase 1: Co-creation (p. 7) and Phase 3: Piloting and Evaluation (p. 12)	Inclusion and exclusion criteria are specified for both breast cancer survivors and healthcare professionals.

	14b	If applicable, eligibility criteria for sites and for individuals who will deliver the interventions (e.g., surgeons, physiotherapists)	Methods – Phase 1: Co-creation (p. 7) and Phase 3: Piloting and Evaluation (p. 12)	Criteria for participating healthcare professionals include relevant clinical experience and digital competence.
Intervention and comparator	15a	Intervention and comparator with sufficient details to allow replication including how, when, and by whom they will be administered. If relevant, where additional materials describing the intervention and comparator (e.g., intervention manual) can be accessed	Methods – Phase 3: Procedure (p. 13).	Describes the SerenApp intervention (8-week digital use) and control (usual care), delivered via mobile app link; both groups complete pre/post assessments.
	15b	Criteria for discontinuing or modifying allocated intervention/comparator for a trial participant (e.g., drug dose change in response to harms, participant request, or improving/worsening disease)	Methods – Phase 3 (pp. 13 -18) and Ethics (p. 18)	Participants may withdraw at any time; discontinuation criteria follow ethical and data protection procedures.
	15c	Strategies to improve adherence to intervention/comparator protocols, if applicable, and any procedures for monitoring adherence (e.g., drug tablet return, sessions attended)	Methods – Phase 3 (p. 16).	Adherence monitored via app use data (frequency, duration, module completion, engagement indicators).
	15d	Concomitant care that is permitted or prohibited during the trial	Methods – Phase 1: Co-creation (p. 7) and Phase 3: Piloting and Evaluation (p. 12). Inclusion/exclusion criteria.	Ongoing psychological or pharmacological treatments are allowed if inclusion criteria are met; usual oncological care continues for all participants.
Outcomes	16	Primary and secondary outcomes, including the specific measurement variable (e.g., systolic blood pressure), analysis metric (e.g., change from baseline, final value, time to event), method of aggregation (e.g., median, proportion), and time point for each outcome	Methods – Phase 3 (pp. 14-18).	Primary outcomes: stress, anxiety, and depression. Secondary outcomes: quality of life, usability, satisfaction, adherence, cost-effectiveness, and cost-utility.
Harms	17	How harms are defined and will be assessed (e.g., systematically, non-systematically)	Methods – Phase 3, Procedure (p. 13).	Adverse events monitored through participant self-reports and researcher follow-up; serious events reported to the ethics committee.
Participant timeline	18	Time schedule of enrollment, interventions (including any run-ins and washouts), assessments, and visits for participants. A schematic diagram is highly recommended (see Figure)	Methods - Study design and setting (pp. 5–6), Figure 1	Study timeline summarized in Figure 1: recruitment (Sept 2026), intervention (8 weeks), and assessments at baseline and post-treatment.
Sample size	19	How sample size was determined, including all assumptions supporting the sample size calculation	Methods – Phase 3, Section 3.1. Sample Size (p.13)	Total sample = 90 participants; power = 80 %; α = 0.05; estimated effect size = 0.5 SD; dropout rate = 15–30 %.
Recruitment	20	Strategies for achieving adequate participant enrollment to reach target sample size	Methods – Phase 3: Procedure (p. 13)	Recruitment coordinated through oncology services in both hospitals, during scheduled follow-up visits; ER contact ensures informed consent and participation.

Methods: Assignment of interventions				
Randomization:				
Sequence generation	21a	Who will generate the random allocation sequence and the method used	Methods – Phase 3: Piloting and Evaluation, Section 3.2 Procedure (p. 13)	The Evaluating Researcher (ER) will generate the random allocation sequence using a table of random numbers to assign participants to intervention or control groups.
	21b	Type of randomization (simple or restricted) and details of any factors for stratification. To reduce predictability of a random sequence, other details of any planned restriction (e.g., blocking) should be provided in a separate document that is unavailable to those who enroll participants or assign interventions	Methods – Phase 3: Piloting and Evaluation, Section 3.2 Procedure (p. 13)	Simple randomization with a 1:1 allocation ratio will be used. No stratification or blocking factors are planned due to the pilot nature of the study.
Allocation concealment mechanism	22	Mechanism used to implement the random allocation sequence (e.g., central computer/telephone; sequentially numbered, opaque, sealed containers), describing any steps to conceal the sequence until interventions are assigned	Methods – Phase 3: Piloting and Evaluation, Section 3.2 Procedure (p. 13)	Allocation will be implemented by the ER using a random number table. The ER and oncology professionals will remain blinded to group allocation to ensure concealment.
Implementation	23	Whether the personnel who will enroll and those who will assign participants to the interventions will have access to the random allocation sequence	Methods – Phase 3: Piloting and Evaluation, Section 3.2 Procedure (p. 13)	Personnel enrolling participants (oncology professionals) and those assigning interventions (ER) will not have access to the randomization sequence to maintain allocation concealment.
Blinding	24a	Who will be blinded after assignment to interventions (e.g., participants, care providers, outcome assessors, data analysts)	Methods – Phase 3: Piloting and Evaluation, Section 3.2 Procedure (p. 13)	Both the Evaluating Researcher (ER) and oncology professionals will be blinded to the group allocation. Participants will not be informed of which arm constitutes the experimental or control condition.
	24b	If blinded, how blinding will be achieved and description of the similarity of interventions	Methods – Phase 3: Piloting and Evaluation, Section 3.2 Procedure (p. 13)	Blinding will be maintained by using a random number table managed solely by the ER, who will not be aware of the allocation sequence. Both groups will receive a digital link to access their respective study materials, minimizing differences in participant experience.
	24c	If blinded, circumstances under which unblinding is permissible, and procedure for revealing a participant's allocated intervention during the trial	Methods – Phase 3: Piloting and Evaluation, Section 3.2 Procedure (p. 13); Ethics and Dissemination (p. 18)	Unblinding will only occur if required for safety reasons or by request of the Ethics Committee. Procedures for unblinding will follow the ethical protocol approved by the Costa del Sol University Hospital Ethics Committee.

Methods: Data collection, management, and analysis				
Data collection methods	25a	Plans for assessment and collection of trial data, including any related processes to promote data quality (e.g., duplicate measurements, training of assessors) and a description of trial instruments (e.g., questionnaires, laboratory tests) along with their reliability and validity, if known. Reference to where data collection forms can be accessed, if not in the protocol	Methods – Phase 3: Piloting and Evaluation, Section 3.3 Study Measures (pp. 14–16)	Data will be collected using validated and reliable instruments: CAEPO (stress), PHQ-9 (depression), HADS (anxiety/depression), EQ-5D-5L (HRQoL), CSRI (service use), SUS (usability), CSQ-8 (satisfaction), and MARS (app quality). Assessors (ER) will be trained in standardized procedures to ensure data quality.
	25b	Plans to promote participant retention and complete follow-up, including list of any outcome data to be collected for participants who discontinue or deviate from intervention protocols	Methods – Phase 3: Procedure and Data Analysis (pp. 16–18)	Retention will be promoted through regular communication, digital reminders, and usability support. Data from participants who discontinue will be included in intention-to-treat analyses using baseline and available follow-up data.
Data management	26	Plans for data entry, coding, security, and storage, including any related processes to promote data quality (e.g., double data entry; range checks for data values). Reference to where details of data management procedures can be accessed, if not in the protocol	Ethics and Dissemination (pp. 18–19)	Data will be anonymized and securely stored in encrypted databases accessible only to authorized researchers. Coding and range checks will be applied to ensure accuracy. Data handling will comply with GDPR (EU 2016/679) and Spanish Organic Law 3/2018 on Data Protection.
Statistical methods	27a	Statistical methods used to compare groups for primary and secondary outcomes, including harms	Methods – Phase 3: Section 3.4 Data Analysis (pp. 16–18)	Quantitative analyses will include descriptive statistics, ANOVA or Kruskal-Wallis tests for group comparisons, and multivariate models (e.g., multilevel regression). Cost-effectiveness and cost-utility analyses will follow international guidelines.
	27b	Definition of who will be included in each analysis (e.g., all randomized participants), and in which group	Methods – Phase 3: Section 3.4.1 Efficacy Analysis (p. 16)	Analyses will follow both intention-to-treat and per-protocol principles. All randomized participants will be analyzed in their originally assigned groups.
	27c	How missing data will be handled in the analysis	Methods – Phase 3: Section 3.4.1 Efficacy Analysis (p. 16)	Missing data will be addressed using multiple imputation techniques where appropriate and by conducting sensitivity analyses to assess robustness.
	27d	Methods for any additional analyses (e.g., subgroup and sensitivity analyses)	Methods – Phase 3: Section 3.4.1 Efficacy Analysis (p. 16–18)	Subgroup and sensitivity analyses will be conducted to evaluate robustness of results and explore cost-effectiveness under varying assumptions.

**Methods: Monitoring**				
Data monitoring committee	28a	Composition of data monitoring committee (DMC); summary of its role and reporting structure; statement of whether it is independent from the sponsor and funder; conflicts of interest and reference to where further details about its charter can be found, if not in the protocol. Alternatively, an explanation of why a DMC is not needed	Not applicable – See Ethics and Dissemination (pp. 18–19)	A Data Monitoring Committee will not be established, as this is a low-risk pilot study involving a non-invasive digital psychological intervention. Trial oversight will be conducted by the principal investigators and research team. The Ethics Committee of Costa del Sol University Hospital will supervise compliance with ethical and data protection standards.
	28b	Explanation of any interim analyses and stopping guidelines, including who will have access to these interim results and make the final decision to terminate the trial	Methods – Phase 3: Data Analysis (pp. 16–18); Ethics and Dissemination (pp. 18–19)	No interim analyses or early stopping guidelines are planned due to the exploratory and pilot nature of the study. Any decision to modify or terminate the trial will be made by the research team in consultation with the Ethics Committee.
Trial monitoring	29	Frequency and procedures for monitoring trial conduct. If there is no monitoring, give explanation	Ethics and Dissemination (pp. 18–19)	Trial conduct will be internally monitored by the research team through regular coordination meetings. The Ethics Committee will oversee compliance with Good Clinical Practice (GCP) and data protection requirements. No independent external monitoring is required given the minimal risk nature of the study.
**Ethics**				
Research ethics approval	30	Plans for seeking research ethics committee/institutional review board approval	Ethics and Dissemination (pp. 18–19)	Ethical approval was obtained from the Research Ethics Committee of the Costa del Sol University Hospital (reference: 002_nov23-PI2-APP Depresión y CaMama), in accordance with the Declaration of Helsinki and Good Clinical Practice (GCP) guidelines.
Protocol amendments	31	Plans for communicating important protocol modifications to relevant parties	Ethics and Dissemination (pp. 18–19)	Any significant protocol modifications (e.g., eligibility criteria, outcomes, analyses) will be communicated to the Ethics Committee, trial registry (ClinicalTrials.gov), and relevant collaborators prior to implementation.
Consent or assent	32a	Who will obtain informed consent or assent from potential trial participants or authorized proxies, and how	Ethics and Dissemination (pp. 18–19); Methods – Phase 3: Procedure (p. 13)	Informed consent will be obtained by the Evaluating Researcher (ER) or oncology professionals during follow-up consultations. Participants will receive detailed written and verbal information about study objectives,

				procedures, and data management before signing consent.
	32b	Additional consent provisions for collection and use of participant data and biological specimens in ancillary studies, if applicable	Not applicable – Ethics and Dissemination (p. 18)	No biological specimens will be collected. Participants consent only to the use of anonymized data for research and publication purposes related to the present study.
Confidentiality	33	How personal information about potential and enrolled participants will be collected, shared, and maintained in order to protect confidentiality before, during, and after the trial	Ethics and Dissemination (pp. 18–19)	All participant data will be pseudonymized, encrypted, and stored in password-protected databases accessible only to authorized researchers. Clinical data will be kept separate from identifying information, in compliance with GDPR (EU 2016/679) and Spanish Organic Law 3/2018.
Ancillary and post-trial care	34	Provisions, if any, for ancillary and post-trial care, and for compensation to those who suffer harm from trial participation	Ethics and Dissemination (p. 19)	Given the non-invasive nature of the digital intervention, no adverse effects are expected. Participants will be referred to their healthcare providers if distress arises. Compensation will follow institutional and national ethical regulations if any harm occurs.

*We strongly recommend reading this checklist in conjunction with the SPIRIT 2025 Explanation and Elaboration and the SPIRIT 2025 Expanded Checklist for important clarifications on all the items. We also recommend reading relevant SPIRIT extensions. See www.consort-spirit.org

Citation: Chan A-W, Boutron I, Hopewell S, Moher D, Schulz KF, et al. SPIRIT 2025 statement: updated guideline for protocols of randomised trials. BMJ 2025;389:e081477. https://dx.doi.org/10.1136/bmj-2024-081477

© 2025 Chan A-W et al. This is an Open Access article distributed under the terms of the Creative Commons Attribution License (https://creativecommons.org/licenses/by/4.0/), which permits unrestricted use, distribution, and reproduction in any medium, provided the original work is properly cited.
